# An N-Ethyl-N-Nitrosourea (ENU)-Induced Dominant Negative Mutation in the JAK3 Kinase Protects against Cerebral Malaria

**DOI:** 10.1371/journal.pone.0031012

**Published:** 2012-02-21

**Authors:** Silayuv E. Bongfen, Ian-Gael Rodrigue-Gervais, Joanne Berghout, Sabrina Torre, Pablo Cingolani, Sean A. Wiltshire, Gabriel A. Leiva-Torres, Louis Letourneau, Robert Sladek, Mathieu Blanchette, Mark Lathrop, Marcel A. Behr, Samantha Gruenheid, Silvia M. Vidal, Maya Saleh, Philippe Gros

**Affiliations:** 1 Department of Biochemistry, McGill University, Montreal, Canada; 2 Department of Medicine, McGill University, Montreal, Canada; 3 Department of Human Genetics, McGill University, Montreal, Canada; 4 Complex Traits Group, McGill University, Montreal, Canada; 5 School of Computer Science, McGill University, Montreal, Canada; 6 Institut de Génomique, Centre National de Génotypage, Evry, France; 7 Department of Microbiology and Immunology, McGill University, Montreal, Canada; 8 The McGill University Health Center, Montreal, Canada; Harvard Medical School, United States of America

## Abstract

Cerebral malaria (CM) is a lethal neurological complication of malaria. We implemented a genome-wide screen in mutagenized mice to identify host proteins involved in CM pathogenesis and whose inhibition may be of therapeutic value. One pedigree (P48) segregated a resistance trait whose CM-protective effect was fully penetrant, mapped to chromosome 8, and identified by genome sequencing as homozygosity for a mis-sense mutation (W81R) in the FERM domain of Janus-associated kinase 3 (Jak3). The causative effect of *Jak3^W81R^* was verified by complementation testing in *Jak3^W81R/−^* double heterozygotes that were fully protected against CM. *Jak3^W81R^* homozygotes showed defects in thymic development with depletion of CD8^+^ T cell, B cell, and NK cell compartments, and defective T cell-dependent production of IFN-γ. Adoptive transfer of normal splenocytes abrogates CM resistance in *Jak3^W81R^* homozygotes, an effect attributed to the CD8^+^ T cells. *Jak3^W81R^* behaves as a dominant negative variant, with significant CM resistance of *Jak3^W81R/+^* heterozygotes, compared to CM-susceptible *Jak3^+/+^* and *Jak3^+/−^* controls. CM resistance in *Jak3^W81R/+^* heterozygotes occurs in presence of normal T, B and NK cell numbers. These findings highlight the pathological role of CD8^+^ T cells and Jak3-dependent IFN-γ-mediated Th1 responses in CM pathogenesis.

## Introduction

Malaria, caused by infection with members of the *Plasmodium* family of parasites, still remains a global health problem, with close to 250 million clinical cases and almost a million deaths occurring each year, mostly in African children [1]. Cerebral malaria (CM) is the most severe complication of *P. falciparum* infection. Although CM develops in less than 1% of *Plasmodium* infected individuals, its sudden onset, rapid progression and limited treatment options (high dose quinine or artemisnin) contribute to an often-lethal outcome. CM is characterized by trapping of parasitized erythrocytes in the host microvasculature including the blood brain barrier (BBB) that triggers a strong inflammatory response *in situ*, vascular damage and hypoxia. Permeability of the BBB leads to seizures, paralysis, unrousable coma and death [2]. The cell and molecular pathways involved in CM pathogenesis are poorly characterized and need to be better understood to identify novel therapeutic targets for intervention. Clinical epidemiological studies in different geographical areas of malaria-endemicity have indicated that the onset, progression and outcome of CM involve a complex interplay between environmental factors, parasite-expressed virulence factors and host genetic factors influencing replication of the parasite or innate or acquired immunity [3], [4], [5]. Genetic studies in humans have pointed to a heritable component to susceptibility to CM (reviewed in [6]), while case-control association studies have suggested a complex and heterogeneous genetic component in CM, including hemoglobin variants (hemoglobinopathies), polymorphisms in cytokine genes or gene promotors, and many others (reviewed by [3], [4]).

The complex genetic component of CM susceptibility has also been investigated in the mouse model for experimental CM caused by infection with *Plasmodium berghei* ANKA (*Pb*A). *P. berghei* infection in mice closely mimics *P. falciparum*-induced CM in humans [7], with susceptible mouse strains (e.g. C57BL/6J, CBA/J) developing an acute cerebral syndrome within 6–7 days characterized by ataxia, paraplegia, seizures and coma leading to uniform lethality by day 8–10 post-infection. Like in humans, studies in mouse mutants bearing inactivating mutations at specific genes have shown that host-driven inflammation plays a central role in CM pathogenesis [2], [8]. Indeed, local production of pro-inflammatory cytokines such as IFN-γ [9], TNF-α [10] and LT-α [11], upregulation of chemokine ligands [12] and cell adhesion molecules [13], as well as sequestration of immune cells (CD4^+^, CD8^+^, NK cells) [12], [14], [15] and parasites [15] in the brain microvasculature have all been shown to play a role in CM. On the other hand, inactivating mutations in genes coding for pro-inflammatory molecules [16], [17], and generation of anti-inflammatory cytokines like IL-10 [14] and IL-4 [18] have been associated with protection from CM.

Genome-wide linkage studies in informative crosses derived from mouse strains showing varying degrees of susceptibility to *P. berghei*-induced CM have detected at least six quantitative trait loci (QTL) - designated *berghei* resistance (*Berr*) loci – as modulating response to infection including survival from acute infection: *Berr1* (chromosome 1), *Berr2* (chromosome 11), *Berr3* (chromosome 9), *Berr4* (chromosome 4), *Berr5* (chromosome 19), and the *Cmsc* locus mapping to the H-2 region of chromosome 17 [19], [20], [21], [22]. The *Berr5* locus co-localizes with three other immune loci, including *Trl-4* (tuberculosis resistance), *Tsiq2* (T-cell secretion of IL-4), and *Eae19* (experimental allergic encephalitis 19) suggesting the possibility of a common genetic effect underlying these phenotypes. Nevertheless, the modest effects of these individual loci, the relatively large size of the chromosomal regions mapping underneath the QTL peaks, and the large number of positional candidates have precluded the positional cloning of the genes involved.

ENU mutagenesis is a powerful experimental tool used to introduce random mutations in the mouse germ-line. Such mutations can be propagated in informative pedigrees, where they can be bred to homozygosity, and where their effect on a given physiological system or host pathway can be investigated. In high throughput screening experiments, such mutations may manifest themselves as rare pheno-deviant pedigrees displaying unique disease-associated phenotypes. The positional cloning of the mutant gene, facilitated by the *de novo* nature of the mutation (absent from the reference sequence), may in turn identify novel proteins that play a role in the specific phenotype and associated pathology. This strategy has been used successfully to identify genes, proteins and pathways in a broad range of disease states, including susceptibility to infections [23], obesity [24], muscle development and function [25], cardiomyopathy [26] and thrombocytopenia [27].

In this study, we implemented a large scale ENU mutagenesis strategy to identify genes that play an important role in the pathogenesis of cerebral malaria. Intravenous infection of C57BL/6J and C57BL/10J mice with 10^6^ *P. berghei*-parasitized erythrocytes is uniformly lethal with all animals developing cerebral symptoms by day 5–6 and succumbing to infection by days 7–10. We searched for recessive mutations that would protect mice form *P. berghei*-induced CM and associated lethality, and that would confer survival to this otherwise lethal infection. We aimed to identify novel protein and biochemical pathways that may constitute novel targets for small molecule inhibition and therapeutic intervention in this lethal infection. In a first example of this screen, we report the identification of a pheno-deviant pedigree that displays segregation of a CM-resistance phenotype. We demonstrate that this resistance is phenotypically expressed as a severe depletion of several immune cell compartments including CD8^+^ T cells, B cells and NK cells, and caused by a mutation in the *Jak3* gene. Resistance to CM in this mutant is associated with an impaired Th1 response, which is concomitant with increased susceptibility to infection with mycobacteria (*M. tuberculosis*), and *Citrobacter*.

## Results

### Identification and characterization of a cerebral malaria resistant ENU mutant

**Figure 1:**
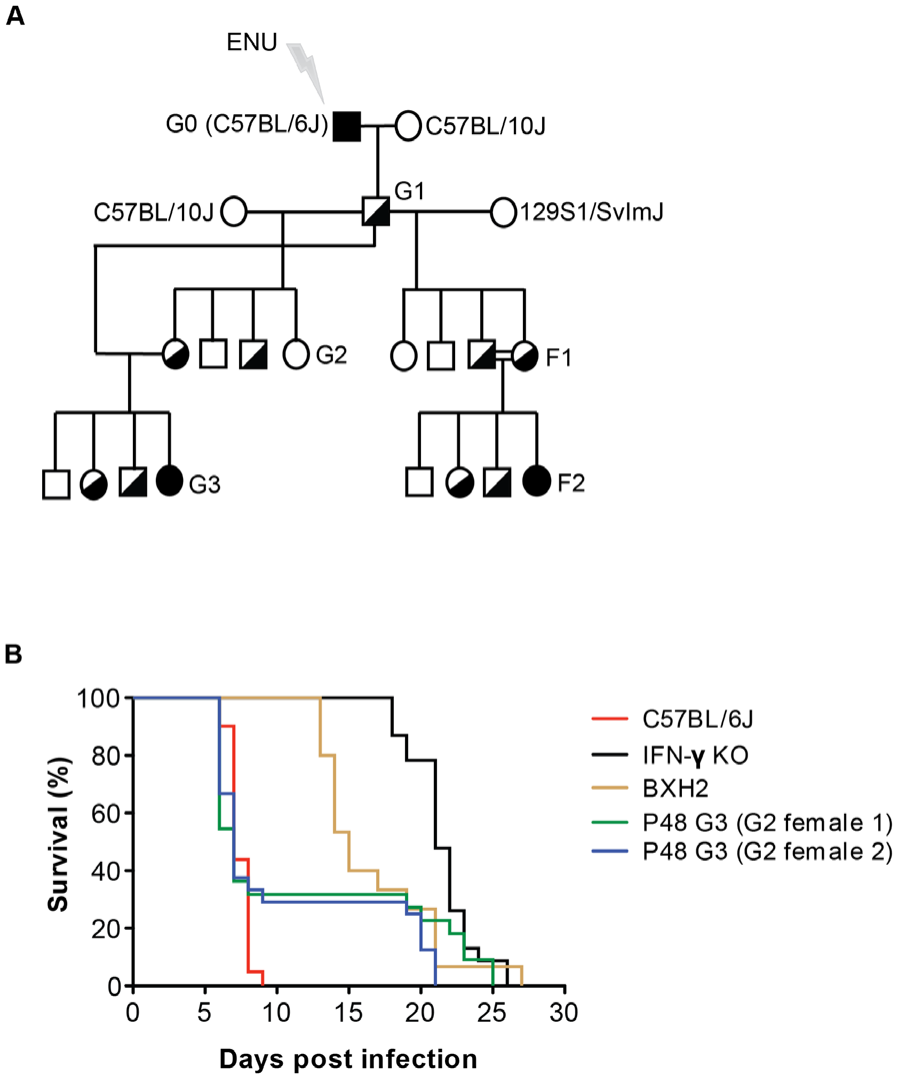
ENU-induced mutation that protects mice against *P. berghei* ANKA-induced cerebral malaria. (**A**) Breeding scheme for the production and identification of ENU-induced recessive mutations that convey protection against cerebral malaria (CM). Details of the breeding strategy are described in “Materials and Methods”. G3 and F2 pedigrees were phenotyped for the presence of animals resistant to *P. berghei*-induced CM. (**B**) Mice were infected with *P. berghei* ANKA (10^6^ *P. berghei* ANKA-parasitized red blood cells, i.v.) and survival was monitored over time for individual pedigree P48 G3s (green and blue lines) derived from independent G2 females and a G1 male, and for susceptible C57BL/6J (B6, red line), and resistant mutant mouse strains bearing loss of function mutations in either the IFN-γ gene KO (IFN-γ KO, black line) or IRF8 (BXH2, brown line). Mice surviving past day 13 post infection were considered to be CM-resistant.

To identify genes, proteins, and cellular pathways important for the pathogenesis of cerebral malaria (CM), we screened pedigrees derived from ENU-mutagenized mice, looking for the appearance of CM-resistant pheno-deviant pedigrees on the otherwise CM-susceptible genetic background of C57Bl/6J (B6). Such pedigrees are believed to segregate protective mutations fixed for homozygosity, and affecting genes that are important for CM pathogenesis including host-driven detrimental effects. In our protocol, mutagenized B6 males were crossed to C57Bl/10J (B10, to facilitate subsequent genetic mapping), and the resulting G1 males were backcrossed to B10 (Figure 1A); the resulting G2 females (two per G1 male) were backcrossed to their G1 father to produce G3 pedigrees where mutations are fixed to homozygosity in 25% of the animals. These G3 pedigrees were infected with *P. berghei* ANKA, and we monitored the presence of pheno-deviant progeny that fail to develop cerebral symptoms and survive this infection. When such positive pedigrees were detected, additional G3 animals from the same G2 females and G1 father were generated and phenotyped to validate the presence of a protective mutation. Screening a total of 3967 G3 mice from 153 pedigrees identified several such pheno-deviant pedigrees. One of these pedigrees, #48 (P48), displayed a fairly high percentage of resistant animals (∼31% resistance), with both G2 females producing CM-resistant offspring (Figure 1B), and was chosen for further analysis.

**Figure 2:**
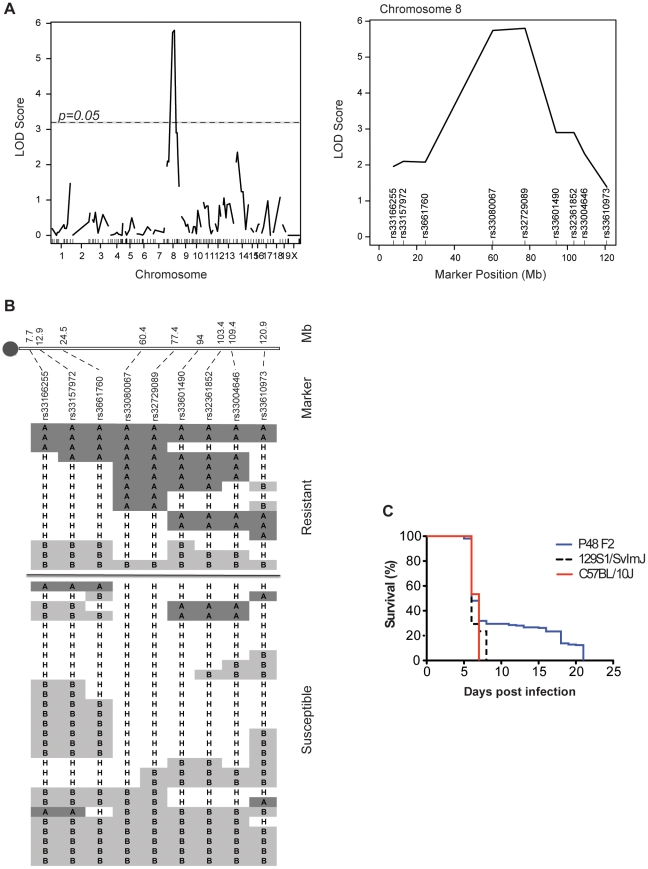
Resistance to cerebral malaria in pedigree 48 maps to the central portion of chromosome 8. Genome-wide linkage analysis of the CM-resistance trait (survival) was conducted in 44 G3 (15 resistant, 29 susceptible) mice from pedigree 48, using polymorphic markers informative for the B6 and B10 progenitors. (**A**) LOD score traces identifying significant linkage to chromosome 8 (p = 0.05, genome-wide significance shown as dotted line); the position of informative markers is shown, including rs33080067 and rs32729089 (LOD score ∼5.8). (**B**) Haplotype analysis of the central portion of chromosome 8 in CM-resistant and CM-susceptible G3 animals from pedigree 48 (A, B6 homozygotes; H, B6/B10 heterozygotes; B, B10 homozygotes) showing exclusion of homozygote B6 haplotypes from the CM-susceptible group. The positions of the markers (in Mb) from the centromere (shown as a grey dot) are shown. (**C**) The G1 male from pedigree 48 was out-crossed to 129S1/SvlmJ to generate an F2 population (n = 211) that was phenotyped for response to *P. berghei* ANKA infection. Survival of F2 mice as well as parental 129S1/SvlmJ and C57BL/10J controls is shown.

A genome-wide scan was carried out in 44 G3 animals from P48 (15 CM-resistant and 29 CM-susceptible) using 131 polymorphic markers informative for B6 and B10. Linkage analysis (using R/qtl) identified a 17 Mb region on the central portion of chromosome 8 (95% Bayesian credible interval: 60.4 Mb–77.4 Mb) as regulating differential CM-resistance in this pedigree, with a logarithm of odds (LOD) score of 5.8 (Figure 2A). Haplotype analysis revealed that, as expected, resistance to CM at this locus was associated with homozygosity for B6-derived alleles (from mutated G0 male), while homozygosity for B10 alleles was associated with CM-susceptibility, and with B6/B10 heterozygotes being present in both resistant and susceptible groups (Figure 2B). These findings suggested that the CM-protective effect of this locus is co-dominant in this cross. The P48 G1 male was also outcrossed to CM-susceptible 129S1 WT females, and the resulting offspring were intercrossed to generate a total of 211 F2 mice, which were phenotyped by infection with *P. berghei* ANKA (Figure 2C). Approximately 25% of these F2 survived the cerebral phase, indicating that the resistance trait associated with P48 is fully penetrant on the distinct genetic background of 129S1. Genotyping of these animals verified that CM resistance was controlled by the chromosome 8 locus, and also showed a co-dominant mode of inheritance of B6 protective alleles in this cross (Supplementary Figure S1).

### Immunological phenotyping of P48 mutants

**Figure 3:**
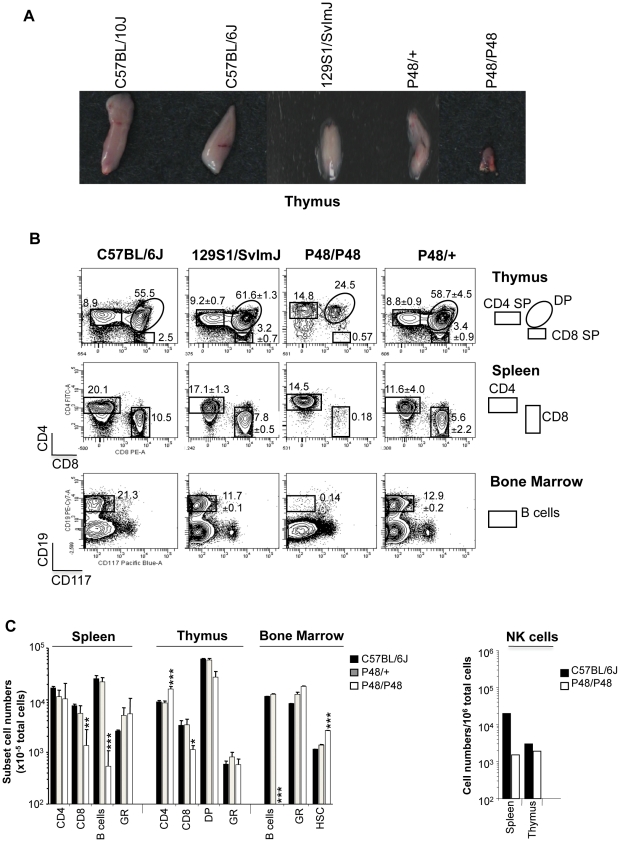
Phenotypic expression of resistance to cerebral malaria in mice from pedigree 48. G3 and F2 mice homozygote (P48/P48) or heterozygote (P48/+) for the B6-derived mutant central chromosome 8 were identified by genotyping, and were subjected to several analyses, along with parental C57BL/6J, C57BL/10J and 129S1/SvlmJ controls. (**A**) Macroscopic examination of thymus from control and mutants showing severely atrophied thymus in homozygote mutants (representative of 5 mice per group). (**B**) FACS density plots of different cell populations in thymus (top), spleen (middle) and bone marrow (bottom) stained for CD4, CD8, CD19, and CD117. The position of the different cell lineages in the scatter plots are identified at the extreme right panel and their numbers are expressed as a percentage (± SE; *n* = 5 mice per group) of total cells in this tissue. (**C**) Flow cytometric analysis of immune cell lineage composition expressed as the absolute number (mean ± SD; *n* = 4–6 mice per group) of CD4^+^ and CD8^+^ single positive (SP), CD4^+^CD8^+^ double positive (DP), B cells (CD19^+^), granulocytes (GR; Gr1^+^), hematopoietic stem cells (HSC; lineage^−^CD117^+^) and NK cells from 10^5^ cells from spleen, thymus and bone marrow. Asterisks (one-way Anova test with Bonferroni post-test) identify significant differences between experimental animals and the C57BL/6J controls: *p<0.05; ** *p*<0.01; *** *p*<0.001. Data are representative of two independent experiments.

To gain insight into the cell population phenotypically expressing the CM-protective mutation, we examined different lymphoid and myeloid organs, and monitored specific cell populations within them in mice fixed for homozygosity for B6-derived alleles at the chromosome 8 locus. Macroscopic examination readily identified severe thymic atrophy in homozygotes (Figure 3A), while heterozygotes were normal, suggesting a possible thymus development defect. FACS analysis further identified a severe depletion of the CD8^+^ T cell compartment in thymus of these mice, while the CD4^+^ T cell compartment appeared unaffected. A similar severe depletion of the CD8^+^ T cell compartment was also detected upon analysis of spleen cells from these mice (Figure 3B), with an additional reduction in NK cells (Figure 3C). On the other hand, studies of bone marrow cells showed a complete absence of CD19^+^ B cells in mutant mice. In all tissues examined, there was no effect noted on the myeloid compartment (Gr1^+^ cells). All alterations in these cell populations were detected only in homozygotes, while heterozygotes showed normal cell numbers, which were similar to C57BL/6J controls.

### Resistance to cerebral malaria is caused by a mutation in the Jak3 kinase

**Figure 4:**
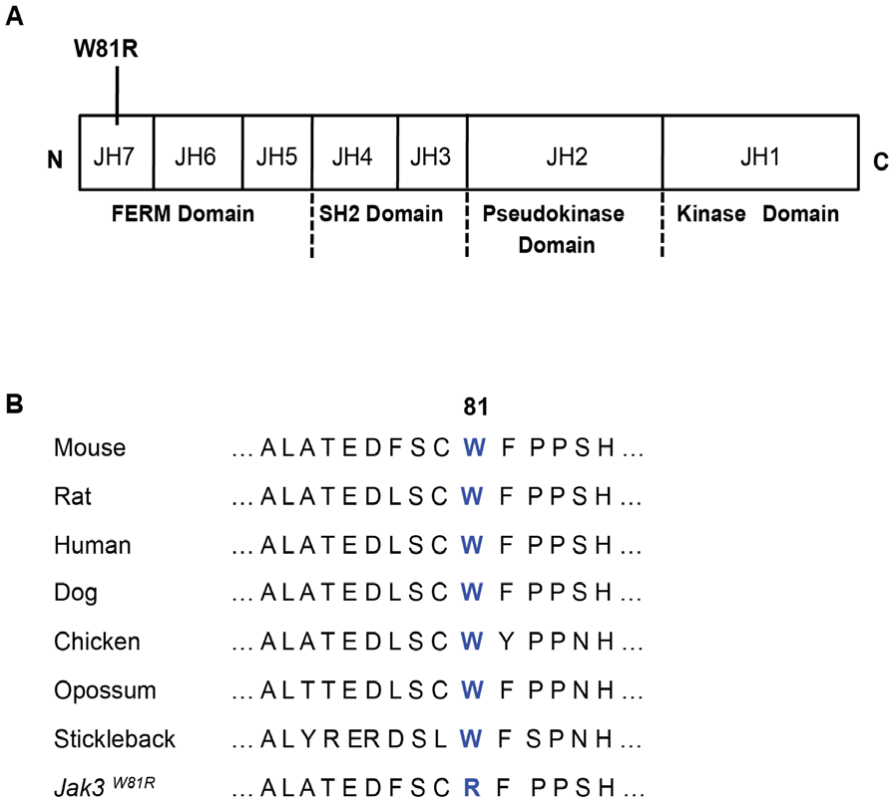
A W81R mutation in the FERM domain of Jak3 causes resistance to cerebral malaria in pedigree 48. (**A**) Schematic representation of the Jak3 protein, showing the 7 Jak Homology (JH) structural domains, and associated functional annotation (below). The position of the W81R mis-sense mutation discovered in pedigree 48 is shown. (**B**) Multiple sequence alignment of the amino terminal portion of Jak3 surrounding W81 shows high conservation across Jak3 relatives (the corresponding species is identified).

**Figure 5:**
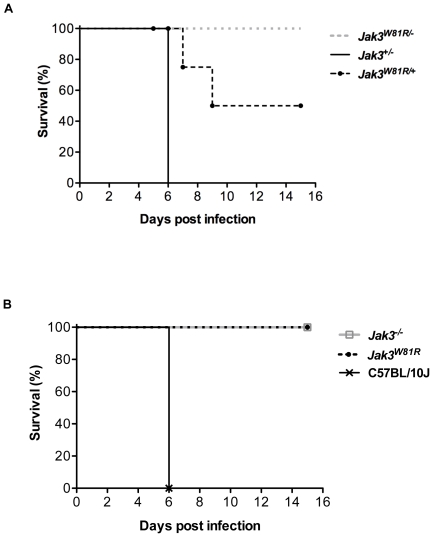
Resistance to cerebral malaria in *Jak3^W81R/−^* compound heterozygotes. *Jak3^W81R^* homozygotes were crossed to a mouse line bearing a null *Jak3* allele (*Jak3^−/−^*) to create the *Jak3^W81R/+^* compound heterozygotes. Single heterozygotes (*Jak3^W81R/+^*, *Jak3^+/−^*; Fig. 5A), homozygotes (*Jak3^W81R^*, *Jak3^−/−^*; Fig. 5B), compound heterozygotes (*Jak3^W81R/−^*, gray line; Fig. 5A) and C57BL/10J controls were infected with 10^6^ *P. berghei* ANKA-parasitized RBCs and monitored for survival. All surviving mice were sacrificed on day 15 post-infection (experimental end-point). The data are representative of 2 independent experiments.

The 17 Mb interval delineating the position of the CM-protective locus on chromosome 8 contains a number of positional candidates that a) have an established role in the immune system, b) are known to be modulated by IFN-γ, or c) show IFN-inducible STAT1 binding sites in their promoter. These include Interleukin 12 receptor beta 1 (IL12rb1; 73.3 Mb), interferon gamma inducible protein 30 (Ifi30; 73.2 Mb), Janus kinase 3 (Jak3; 74.2 Mb), heme oxygenase (decycling) 1 (Hmox1; 77.6 Mb) and unc-13 homolog A (Unc13a; 74.1 Mb). Whole genome sequencing of genomic DNA from P48 mutant homozygotes was undertaken, and candidate variants in the above-mentioned genes that were absent from the B6 reference sequence were further validated by re-sequencing. This analysis revealed a unique T-to-A transversion in exon 2 of the *Jak3* gene, which causes a W81R substitution in the Band 4.1/Ezrin/Radixin/Moesin (FERM) homology domain at the N-terminus of the Jak3 protein (Figure 4). The FERM domain is involved in mediating interactions of Jak3 with different cytokine receptors in immune cells. The tryptophan at position 81 is absolutely conserved in Jak3 relatives from different species, suggesting a conserved structural/functional role of this residue in Jak3 activity. To confirm that the CM-resistance in P48 mutants was caused by a mutation in *Jak3*, we carried out complementation testing using *Jak3^−/−^* null mice (B6.129S4-Jak3^tm1Ljb^; MGI database, [28]). These animals have previously been shown to have atrophied thymuses, as well as low numbers of splenic CD8^+^ T cells, NK cells and a defect in B cell maturation [28]. Compound (*Jak3^W81R/−^*) heterozygotes were generated by crossing *Jak3^W81R^* homozygotes to *Jak3^−/−^* null mice, and these together with *Jak3^W81R/+^* and *Jak3^+/−^* heterozygotes were phenotyped for susceptibility to *P. berghei*-induced CM. Compound *Jak3^W81R/−^* heterozygotes were found to be as CM-resistant as *Jak3^W81R^* homozygotes and as *Jak3^−/−^* null animals, confirming that the *Jak3^W81R^* mutation is indeed responsible for protection against CM in P48. On the other hand, *Jak3^+/−^* heterozygotes were as susceptible to *P. berghei* induced CM as the B10 WT controls. Finally, we noted that 50% of *Jak3^W81R/+^* heterozygotes were resistant to CM, in agreement with haplotype analyses of the original G3 animals. This observation together with the uniform susceptibility of *Jak3^+/−^* heterozygotes, confirmed the co-dominant mode of inheritance of the *Jak3^W81R^* mutation, and suggested that it may have a dominant negative effect (Figure 5).

### Splenocytes from infected B10 WT mice restore CM susceptibility to *Jak3^W81R^* homozygous mutant mice

**Figure 6:**
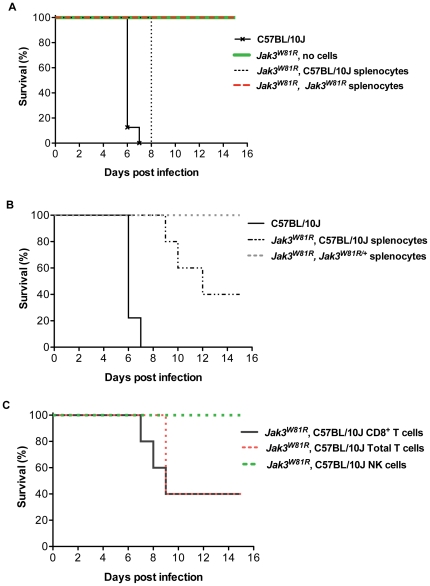
Cell transfer experiments to induce susceptibility to cerebral malaria in *Jak3^W81R^* mice. Wild type C57BL/10J (B10) mice, *Jak3^W81R^* homozygote mutants (Fig. 6A), or *Jak3^W81R^* homozygote mutants having received the indicated cell populations (20 million total splenocytes or 5 million each of the indicated cell population; 2 hrs prior to infection); (Fig. 6A, 6B, 6C) were infected i.v. with 10^6^ *P. berghei* ANKA-parasitized RBCs, and survival from infection was monitored. Untreated wild type B10 and *Jak3^W81R^* mutants were used as susceptible and resistant controls, respectively. All surviving mice were sacrificed on day 15 post-infection (experimental end-point). Data represent 2 independent experiments.

Sequestration of parasitized red blood cells at the brain microvasculature together with local inflammatory response *in situ*, have been shown to be necessary for development and progression of *P. berghei*-induced CM [15], [29]. We aimed to establish in cell transfer experiments, which of the immune cell population(s) missing from the *Jak3^W81R^* mutant mice may be involved in CM pathogenesis, and which absence results in a CM-protective effect. First, total splenocytes from normal B10, from *Jak3^W81R/+^* heterozygote or from *Jak3^W81R^* homozygote mutants, harvested 5 days following infection with *P. berghei* ANKA, were transferred into *Jak3^W81R^* homozygotes. Two hours later, recipient and control un-treated mice were infected with *P. berghei* and the effect of cell transfer on appearance of CM-associated phenotypes and lethality were monitored. The transfer of total spleen cells from wild type B10 controls into *Jak3^W81R^* mutants eliminated CM-resistance in these animals and they succumbed quickly in the cerebral phase of the disease, just like un-treated wild type controls (Figure 6A and 6B). On the other hand, the transfer of total spleen cells from *Jak3^W81R^* homozygote and, interestingly, from *Jak3^W81R/+^* heterozygotes into *Jak3^W81R^* homozygote mutants had no consequence on the CM-resistance trait of these mice (Figure 6A and 6B). To determine which of the absent spleen cell populations is associated with CM-resistance in *Jak3^W81R^* mutants, we carried out further cell fractionation and transfer experiments (Figure 6C). While the transfer of NK cells from infected B10 mice into *Jak3^W81R^* mutants had no effect on CM-resistance, the transfer of either total T cells or of CD8^+^ T cells caused a similar and significant decrease in survival time following *P. berghei* infection of the transferred animals, with 50% of the transferred mice succumbing by day 9. Together, these results indicate that CD8^+^ T cells are critically important for the pathogenesis of *P. berghei*-induced CM, and that their absence in the *Jak3^W81R^* homozygous mutants is in part responsible for the CM-resistance phenotype of these mice.

### *Jak3^W81R^* mutant mice are susceptible to infection with *Mycobacterium* and with *Citrobacter*

**Figure 7:**
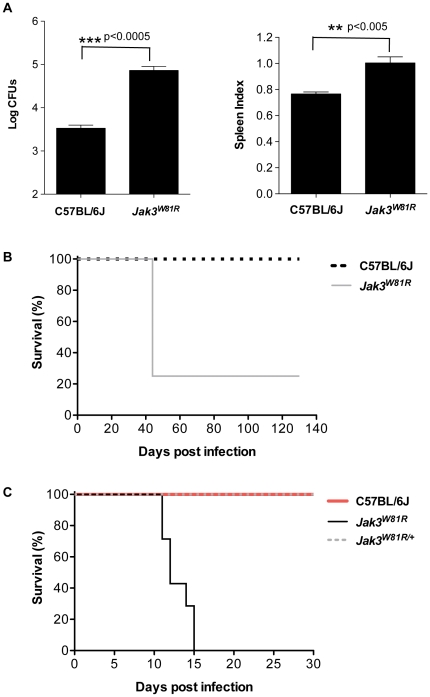
The *Jak3^W81R^* mutation confers susceptibility to infection with different bacterial pathogens. (**A**) Control (C57BL/6J) and *Jak3^W81R^* homozygote mutants were infected with 5×10^4^ colony-forming units (CFUs) of *Mycobacterium bovis* (BCG), and 6 weeks later, mice were sacrificed and the degree of infection was assessed by determination of spleen CFUs (left) and splenomegaly (spleen index; right). (**B**) *Jak3^W81R^* homozygotes and B6 controls were infected by aerosol inoculation of 50 CFUs/lung of *Mycobacterium tuberculosis* H37Rv, and monitored for survival. (**C**) *Jak3^W81R^* homozygotes, *Jak3^W81R/+^* heterozygotes and B6 controls were infected by oral gavage with 3×10^8^ CFUs of *Citrobacter rodentium* strain DBS100. Mice were monitored for survival for 30 days post infection. Data represent two independent experiments.

Pro-inflammatory Th1 cytokines (IFN-γ, TNF-α and LT-α) play an important role in CM pathogenesis, and inactivating mutations in these molecules have a protective effect against *P. berghei*-induced CM. *Jak3^W81R^* mutant mice lack CD8^+^ splenic T cells, and total spleen cells from *Jak3^W81R^* mutants do not produce IFN-γ in response to activation along the Th1 pathway (data not shown). On the other hand, CD4^+^ and CD8^+^ T cells, as well as Th1 cytokines produced by these and other cells are absolutely required for protection against intracellular pathogenic mycobacteria [30]. Therefore, we evaluated the response of *Jak3^W81R^* mice to infection with mycobacteria (Figure 7). Mice were infected with low dose *M. bovis* BCG and bacterial replication was measured 6 weeks following infection (Figure 7A). *Jak3^W81R^* mutants showed splenomegaly and increased spleen bacterial counts (by a factor of 10 fold) compared to control B6 mice. Likewise, a large proportion (>75%) of *Jak3^W81R^* mutants succumbed within 45 days following aerosol infection with virulent *M. tuberculosis* H37Rv, while all of the control B6 mice survived over the same period (Figure 7B). Together, these results indicate that inactivation of *Jak3* kinase causes susceptibility to mycobacterial infection. Independently, it is known that effective protection against enteropathogenic bacteria such as *Citrobacter rodentium* requires intact CD4^+^ T and B lymphocyte compartments [31], and is associated with a robust production of Th1 cytokines such as IFN-γ and TNF-α [32], [33]. Therefore, we assessed the response of *Jak3^W81R^* homozygote and *Jak3^W81R/+^* heterozygote mutants to infection with *C. rodentium* (Figure 7C). Inactivation of *Jak3* caused a dramatic increase in susceptibility to infection, leading to progressive mortality within 15 days of infection, compared to B6 and *Jak3^W81R/+^* heterozygotes which developed transient disease symptoms but survived the infection. These results indicate that full activation of *Jak3* is required for effective Th1-driven host response to infections with extracellular and intracellular pathogens.

## Discussion

Cerebral malaria has a devastating impact on global health. The sudden appearance, rapid progression, and irreversible nature of the CM pathology together with the paucity of treatment options (limited to high dose parenteral administration of quinine and artemisinin derivatives [34]), underlie the high rate of mortality and morbidity associated with CM. Therefore, there is a desperate need for novel therapeutic interventions. CM has a complex pathology that involves multiple host tissues and physiological pathways, including the eyrthroid replicative niche of the parasite, the structure and secretion products of endothelial cells of the microvasculature, and different cell populations, cytokines and associated pathways of the innate and acquired immune system, the activity of which is triggered by variable parasite virulence determinants and further modulated by intrinsic genetic factors of the host [3], [4], [5]. A better characterization of the molecular pathways involved is required to identify novel targets for drug development and therapeutic intervention in CM. Studies in human clinical cases of CM and experiments in mouse models of *P. berghei*-induced CM have identified excessive TNF-a and IFN-g-driven inflammatory response as a key determinant in CM pathogenesis [2]; novel strategies to blunt this response have shown promise in the prevention and treatment of CM [35], [36], [37], [38], [39]. We have implemented a genome-wide mutagenesis screen in mice to systematically identify genes and proteins that mediate pathological inflammatory responses during *P. berghei* infection *in vivo*, and whose pharmacological or genetic inactivation may protect from CM.

We report on the first mutant identified in this screen. Linkage analyses, genomic DNA sequencing, and complementation studies in double heterozygotes have established that CM protection in this mutant is caused by a mutation (W81R) in the amino-terminal FERM domain of Jak3 (Janus kinase 3; *Jak3^W81R^*). Jak3 is a cytosolic tyrosine kinase expressed primarily in the hematopoietic system that plays a critical role in: a) the ontogeny of different myeloid and lymphoid cells, and b) the response of these cells to stimulation by different cytokines. Jak3 interacts with the common gc chain of type 1 and 2 cytokine receptors, which includes IL-2, IL-4, IL-7, IL-9, IL-15 and IL-21. This interaction causes recruitment and phosphorylation of STAT family members to trigger downstream transcriptional responses in cells expressing such receptors [40]. In humans, loss of either JAK3 or IL2RG (which codes for the common gc chain) causes autosomal and X-linked T^−^B^+^ SCID, respectively [41]. These patients lack T cells and NK cells, have normal numbers of immature and poorly functional B-lymphocytes, a clinical picture in agreement with the established roles of IL-7 (T cell development), IL-2 (peripheral T cell homeostasis and antigen-driven T-cell expansion), IL-15 (differentiation of NK cells), and IL-4 (B-cell maturation and isotype switching) in which associated signaling is impaired in JAK3 mutants [41]. *Jak3^W81R^* homozygote mice showed a phenotype that overlaps T^−^B^+^ SCID in humans, and displayed an atrophied thymus, a low number of thymic and splenic CD8^+^ T cells and NK cells, as well as a near absence of B cells in the bone marrow. CD4^+^ T cells were present in normal numbers but appeared anergic and did not produce IFN-g in response to simulation with PMA or ionomycin (under conditions of Th1 polarization) (Figures 3 and 4). This immune phenotype closely resembled that of previously described Jak3 knockout mice [28].

What is the mechanism underlying protection against *P. berghei*-induced CM in *Jak3^W81R^* homozygotes? IFN-g plays a critical role in initiating and amplifying pathological inflammatory response during CM, and mouse mutants lacking the IFN-g gene are protected against *P. berghei*-induced CM (Fig. 1B, [42], [43]). Although the dominant cell type(s) responsible for early production of IFN-g *in vivo* during *P. berghei* infection has been debated, NK cells, CD4^+^ and CD8^+^ T cells have all been implicated [44], [45], and all three populations are affected in *Jak3^W81R^* homozygotes. In the case of NK cells, results either supporting or excluding a role for these cells in CM pathogenesis have been published. In one study, depletion of NK cells failed to alter the appearance of cerebral symptoms or the outcome of CM in *P. berghei* infected mice [43], while another study found that IFN-g secretion by NK cells was important for recruitment of CXCR3^+^ CD4^+^ and CD8^+^ T cells to the brain and development of cerebral disease [43], [44]. On the other hand, cell depletion and cell transfer experiments *in vivo* have shown that IFN-g production by CD4^+^ and CD8^+^ T cells can both contribute to CM pathogenesis [12], [46], [47], [48]. Results from our adoptive transfer studies in *Jak3^W81R^* homozygotes (Figure 6) provide additional insight into this question. We observed that: a) total spleen cells from C57BL/10J mice were the only cell population that could fully restore CM-susceptibility in the mutants; b) total T cells and purified CD8^+^ T cells had a similar effect and caused partial but significant reversion to CM susceptibility in *Jak3^W81R^* animals; c) transfer of purified wild type NK cells had no impact on the CM resistance of the *Jak3^W81R^* mutants. These results strongly suggest that CD8^+^ T cells are the major cell type contributing to CM pathogenesis, although other spleen cell populations or other cell:cell interactions, for example T cell dependent NK cell activation [49], appear to be required to observe the full effect. Nevertheless, our results clearly establish a role of the Jak3 kinase in the pathogenesis of cerebral malaria. This participation may reflect the function of Jak3 in the ontogeny of cell populations (NK cells, CD8^+^ T cells) that produce IFN-g and other soluble mediators of the pathological inflammatory response that are absent in the *Jak3^W81R^* mutant. The protective effect of *Jak3^W81R^* may additionally involve inhibition of gc chain-dependent cytokine receptor signaling in other cell types, whose ontogeny is not affected by the *Jak3* mutation. Nevertheless, our results suggest that pharmacological inhibition of Jak3 may be of therapeutic value in CM. Several small molecule Jak3 inhibitors have been developed and are undergoing clinical evaluation for inflammatory conditions such as rheumatoid arthritis, psoriasis and several autoimmune conditions including autoimmune encephalitis, and rejection of organ transplants [50], [51]. Our findings raise the interesting possibility that Jak3 inhibition by some of these molecules may represent a novel strategy for intervention in clinical cases of CM, a proposition that can be tested experimentally.

An intriguing finding of our study is the intermediate CM-resistance phenotype characteristic of *Jak3^W81R/+^* heterozygotes, with a proportion of these animals either succumbing late in the cerebral phase or completely surviving the cerebral phase. This was first noticed in haplotype analyses of G3 mice of pedigree 48, with animals heterozygote for the chromosome 8 markers being found in both the CM-resistant and CM-susceptible groups (Figure 2B), and subsequently verified during *P. berghei* infection of genotyped *Jak3^W81R/+^* hetrozygotes (Figure 5). The effect is not caused by the genetic background of the animals and is specific for *Jak3^W81R/+^* heterozygosity, as is seen when the mutation is introduced onto either B6/B10 or B6/B10-129S1 mixed genetic backgrounds (Figures 2B, S1). The cellular and molecular basis of co-dominance of the *Jak3^W81R^* mutation is intriguing. It could be explained either by a partial loss of Jak3 function in a dosage dependent pathway or by a specific dominant negative effect of the *Jak3^W81R^* allele. The observation that mice heterozygote for a null *Jak3* mutation (*Jak3^+/−^*) are as susceptible to CM as wild type B6 controls clearly argues for the latter possibility. Interestingly, immunophenotyping results of *Jak3^W81R/+^* heterozygotes show that CM protection in these animals is not associated with alterations in the numbers of NK, T and B lymphocytes, which are all present at normal levels when compared to controls (Figures 3B, 3C). Normal production of IFN-g in response to PMA and ionomycin stimulation under Th1 polarization assay conditions is also seen in *Jak3^W81R/+^* heterozygotes (data not shown). This suggests the possibility of a more subtle dominant negative effect of *Jak3^W81R^* on the biochemical properties of Jak3 in cytokine signaling, and that would nevertheless be critical for establishing the inflammatory process during CM. Such a mechanism could take place in the context of sufficient Jak3 activity that would a) allow seemingly normal maturation of different immune cell lineages (NK, B, T cells), but b) not be sufficient to mediate appropriate signaling during an acute inflammatory situation such as CM. The inability of transferred *Jak3^W81R/+^* heterozygote spleen cells to modify CM-resistance of *Jak3^W81R^* homozygotes agrees with such a model, with partial CM-protection in *Jak3^W81R/+^* heterozygotes being linked to an intrinsic cell autonomous defect of *Jak3^W81R/+^* T/B/NK cells which are present in normal numbers in these mice. Finally, a similar scenario has been previously proposed to account for incomplete penetrance and/or partial expressivity of the human SCID phenotype caused by homozygosity for loss of function *JAK3* mutations in certain familial cases [52].

What would be the molecular basis of a dominant-negative effect of W81R on Jak3 function? Ligand-induced oligomerization of cytokine receptors and associated Jak3 kinases may position wild type and mutant Jak3 variants in close proximity in a signaling complex. In this context, inter-molecular dominant negative effects of gain-of-function Jak3 alleles such as W81R may alter the function of the wild type protein expressed in the same cell. W81 maps in the amino-terminal FERM domain, and several FERM domain mutations have been reported in SCID patients, including M1V, A58P, Del58A, 203DelG, Y100C, D169E and P151R [41], [53], [54]. The study of these and other site-directed FERM domain mutants indicate that this domain plays a key role in multiple aspects of Jak3 function ([54], reviewed in [55]). It is required for membrane targeting and for interaction with the gc chain of cytokine receptor [54], [56]. It also acts as a positive regulator of Jak3 kinase activity: it physically interacts with the JH1-JH2 kinase domain to stimulate both ATP binding and tyrosine phosphorylation [54]. Such interactions may be critical in the early cross-phosphorylation of Jak kinases that normally precedes phosphorylation of neighboring substrates [57]. A dominant negative effect of W81R could possibly act through inhibition of these early cross-phosphorylation events in heterodimers containing both wild type and mutant variants. Jak3 kinase activity is modulated by interaction with several proteins including JAB (Jak binding proteins), CIS, SOCS, SSI, STAM, PIAS and others [41], [58]. A dominant negative effect of W81R may involve stabilization of an inhibited state following interaction of wild type and or mutant variants with these modulators. Additional biochemical studies will be required to elucidate the molecular mechanism of the W81R dominant negative effect.

Finally, although the full blown T^−^/B^+^ SCID disease is caused by complete loss of JAK3 function in humans (homozygosity or compound heterozygosity for mutant variants), our findings with the mouse *Jak3^W81R^* allele suggest that heterozygosity for dominant negative human *JAK3* mutations may cause partially impaired immune response. Although immunodeficiency associated with heterozygosity for a *JAK3* mutation has rarely been reported (most of the *JAK3* mutations identified to date are homozygous or compound heterozygous)[45], [57], our findings raise the possibility that a spectrum of mild immunodeficiency associated with heterozygote *JAK3* mutations may be broader than previously suspected.

## Materials and Methods

### Ethics statement

All mice were maintained under pathogen-free conditions and handled according to the guidelines of the Canadian Council of Animal Care. The experimental protocol (Protocol number 5287) was approved by the ethics committee of McGill University.

### Mice and parasites

Wild type (WT) C57BL/6J (B6), C57BL/10J (B10) and 129S1/SvImJ (129S1) mice were purchased from the Jackson laboratories (Bar Harbor, Maine, USA). ENU-mutagenized mice were bred at the animal facility of the Goodman Cancer Centre, McGill University. Mice were maintained under pathogen-free conditions and handled according to guidelines of the Canadian Council on Animal Care. *P. berghei* ANKA parasites were a kind gift from Dr. Mary M. Stevenson, Montreal General Hospital Research Institute, McGill University. Parasites were maintained as frozen stocks at −80°C, and passaged weekly in B10 mice (donor mice). Infected B10 mice were monitored daily for parasitemia and when parasitemia reached 4–7%, mice were bled, and the blood was diluted in 1× phosphate-buffered saline (PBS) for infection of ENU-mutagenized mice.

### ENU Mutagenesis and breeding

Twenty 8-week-old WT B6 male mice were mutagenized by intraperitoneal injection of a fractionated dose of 3×90 mg/kg of ENU at 1-week intervals. After recovery of fertility (8–15 weeks post treatment), treated males were used in a breeding scheme designed to uncover recessive mutations as previously described [59]. Briefly, treated males (G0) were bred to WT B10 females to generate G1 animals, which are heterozygous for mutations across their genome. G1 males were crossed to B10 females to generate G2 animals, each of which has a 50% chance of inheriting any single mutation carried by their G1 father. Two G2 females were backcrossed to their G1 father to generate G3 animals, about a quarter of which were expected to be homozygous for mutations carried by the G1 male. In order to introduce a higher degree of polymorphism in the offspring to facilitate genetic mapping, G1 males from pedigrees with a confirmed heritable resistance trait (after phenotyping of G3 animals) were out-crossed to 129S1 female mice to generate F1 animals. F1 mice were intercrossed to generate F2 animals, 25% of which were expected to carry the mutation from the G1 male fixed to homozygosity (Figure 1A).

### Infection with *Plasmodium berghei* ANKA

G3 and F2 mice at ≥7 weeks of age were infected intravenously (i.v.) with 10^6^ *P. berghei* ANKA-parasitized erythrocytes (obtained from parasite donor mice), and were monitored 2–3 times daily for the appearance of characteristic neurological symptoms, for weight loss and for survival. Mice that survived greater than 13 days post infection with no neurological symptoms were considered to be resistant to cerebral malaria. B10 and 129S1 mice were used as susceptible controls in all experiments, while IFN-γ knockout (KO) mice (on a B6 background) were used as resistant controls.

### DNA preparation and Genetic Mapping

Tail biopsies were obtained from all mice, and genomic DNA was isolated by a standard procedure using proteinase K digestion and phenol/chloroform extraction, as previously described [60], and DNA samples were diluted to 20 ng/µl in distilled H_2_O for genotyping. Genome scanning was performed at the McGill University and Genome Quebec Innovation Centre (Montreal, Qc, Canada), using DNA samples from 15 resistant and 29 susceptible G3 mice from pedigree 48 (P48), and the massArray platform from Sequenom with a panel of 131 B6/B10 polymorphic markers (SNPs) distributed across the genome. Mapping data were analyzed with the R/qtl software version 2.10.1. The binary model was used, and LOD scores calculated using survival as a phenotype. The cutoff for genome-wide significance (*p*<0.05) was 3.23. Further linkage analysis was conducted in 211 additional (P48 X 129S1) F2 mice (see Figure 1A) genotyped for microsatellite markers (Mouse Genome Informatics Database; www.informatics.jax.org) informative for B6 and 129S1 progenitors. Genotyping was carried out by a standard PCR-based method using [a-^32^P] dATP labeling, and resolution on denaturing 6% polyacrylamide gels.

### Genome Sequencing and analysis

The genome of the P48 mutant homozygotes was sequenced using whole-genome shotgun sequencing with 8 lanes of the Illumina GAII sequencer, yielding 133 million 76bp reads (9.1 Gbases in total, corresponding to a 3× coverage of the genome). Sequence extraction and base calling was done using Illumina Pipeline software. Reads were trimmed at five base running median quality value Q = 20 and aligned to the reference genome (mm37) using Bowtie (PMID: 19261174) and BWA (PMID: 19451168). Variant calling was performed using SamTools (PMID: 19505943) and results from both mapping methods were merged after variant calling, producing a total of 350,607 candidate variants. After filtering out non-homozygous and low coverage SNPs (less than 3×) and retaining only coding non-synonymous/non-sense mutations using the ENSEMBL's variant effect predictor, this number was reduced to only 831 variant candidates. Only 6 of those were located in the 17 Mb region of chromosome 8 to which the mutation had been mapped, including 2 candidate mutations in the *Jak3* gene.

### Immunophenotyping

Following isolation of cells from different tissues (spleen, thymus, bone marrow, lymph nodes and blood), the cells were surface stained with appropriate dilutions of antibodies (determined from titration experiments), for 20 minutes in the dark at 4°C, fixed in PBS containing 1% formaldehyde and stored at 4°C in the dark until FACS analysis (performed within 24 h). The following anti-mouse monoclonal antibodies were used: FITC anti-CD4 (RM4-5), PE anti-CD8a (53-6.7), PECy7 anti-CD19 (1D3), APC anti-CD11c (HL3), APCCy7 anti-GR1 (RB6-8C5), V450 anti-CD117 (2B8) (all from BD Pharmingen); PerCPCy5.5 anti-F4/80 (BM8), PerCPCy5.5 anti-CD3e (145-2C11) and eFluor 450 anti-CD11b (MJ7/18) (all from eBioscience). A minimum of 10^5^ cells was collected by FACS for each tissue sample. Data analysis was performed using FACS DiVa version 6.0 software. Initial gating of each sample set used a forward scatter (FSC)-area versus an FSC-height plot to gate out cell aggregates. Immune cells were isolated, and the different cell populations stained with various antibodies (anti-CD3, -CD8, -CD4, -CD19, -CD11c, -CD117) and analyzed by flow cytometry.

### Adoptive transfer experiments

Adoptive transfer was carried out as previously described [12]. Briefly, 8- to-10-week-old wild type (C57BL/10J; B10) or P48 homozygous mutant (P48/P48) mice were injected i.v. with 10^6^ *P. berghei* ANKA-parasitized erythrocytes (RBC). Five days later, spleens were collected in RPMI-3% FBS, and single cell suspensions of viable cells were prepared. Cells were washed in RPMI-3% FBS by centrifugation, and RBC were lysed by re-suspending the final pellet in red blood cell lysis buffer (Sigma), and incubating for 1 minute at RT. Cells were washed again twice as before and counted. CD8^+^ T cells, total T cells or NK cells were purified from infected B10 WT splenocytes by magnetic cell sorting (MACS; Miltenyi) according to the manufacturer's instructions. 5 million CD8^+^ T cells, total T cells or NK cells from B10 WT infected spleens, or 20 million total WT or mutant splenocytes were transferred i.v. into P48/P48 mutant animals. Two hours later, control and reconstituted mice were infected with 10^6^ *P. berghei* ANKA parasites and were monitored for appearance of cerebral symptoms and for overall survival.

### Infection with *Mycobacterium bovis* (BCG) and *Mycobacterium tuberculosis*

Single cell suspensions of *Mycobacterium bovis* BCG (strain Montreal) was prepared for *in vivo* infections as previously described [61]. Briefly, 5×10^4^ colony-forming units (CFUs) were inoculated intravenously into 8–12 week-old mice. Six weeks after infection, mice were sacrificed, weighed and the spleen CFUs were determined by homogenization and plating on Dubos oleic agar base. The level of BCG infection was defined as the logarithm of the mean number of viable BCG recovered from spleens. The spleen index was defined as the square root of the spleen weight (×100) divided by the body weight. For *Mycobacterium tuberculosis* (Mtb) infection, 8–12 week-old mice were infected with 50 CFUs/lung of M. tuberculosis H37Rv by the aerosol route, and survival was monitored.

### Infection with *Citrobacter rodentium*

Mice were infected at four weeks of age with *Citrobacter rodentium* strain DBS100. *C. rodentium* was grown overnight in 3mL Luria-Bertani (LB) broth shaking at 37°C. Mice were infected by oral gavage of 0.1 mL of the overnight culture containing 3×10^8^ CFUs. Following infection with *C. rodentium*, the mice were monitored daily for 30 days post-infection. When any mouse became moribund or reached a clinical endpoint of infection (20% body weight loss, hunching and shaking, inactivity, ruffled fur, anal prolapse, overtly bloody stool, bleeding from the anus and body condition score <2), it was immediately euthanized.

## Supporting Information

Figure S1 **Haplotype map of F2 mice from pedigree 48 for the central portion of chromosome 8 (51.9–84 Mb).** F2 mice generated by crossing the G1 male to 129S1 progenitors were genotyped for microsatellite markers (Mouse Genome Informatics Database; www.informatics.jax.org) in the *51.9–84 Mb* interval, and were phenotyped for resistance and susceptibility to *P. berghei* induced CM. Each row represents the haplotype (A, homozygote B6; H, heterozygote; B, homozygote 129S1) of an individual mouse for the indicated polymorphic markers.
(TIF)

## References

[1] WHO website http://www.who.int/features/factfiles/malaria/en/index.html.

[2] Hunt NH, Grau GE (2003). Cytokines: accelerators and brakes in the pathogenesis of cerebral malaria.. Trends Immunol.

[3] Bongfen SE, Laroque A, Berghout J, Gros P (2009). Genetic and genomic analyses of host-pathogen interactions in malaria.. Trends Parasitol.

[4] Kwiatkowski D (2000). Genetic susceptibility to malaria getting complex.. Curr Opin Genet Dev.

[5] Weatherall DJ (2008). Genetic variation and susceptibility to infection: the red cell and malaria.. Br J Haematol.

[6] Kwiatkowski DP (2005). How malaria has affected the human genome and what human genetics can teach us about malaria.. Am J Hum Genet.

[7] de Souza JB, Hafalla JC, Riley EM, Couper KN (2010). Cerebral malaria: why experimental murine models are required to understand the pathogenesis of disease.. Parasitology.

[8] Miu J, Mitchell AJ, Muller M, Carter SL, Manders PM (2008). Chemokine gene expression during fatal murine cerebral malaria and protection due to CXCR3 deficiency.. J Immunol.

[9] Grau GE, Heremans H, Piguet PF, Pointaire P, Lambert PH (1989). Monoclonal antibody against interferon gamma can prevent experimental cerebral malaria and its associated overproduction of tumor necrosis factor.. Proc Natl Acad Sci U S A.

[10] Grau GE, Fajardo LF, Piguet PF, Allet B, Lambert PH (1987). Tumor necrosis factor (cachectin) as an essential mediator in murine cerebral malaria.. Science.

[11] Engwerda CR, Mynott TL, Sawhney S, De Souza JB, Bickle QD (2002). Locally up-regulated lymphotoxin alpha, not systemic tumor necrosis factor alpha, is the principle mediator of murine cerebral malaria.. J Exp Med.

[12] Campanella GS, Tager AM, El Khoury JK, Thomas SY, Abrazinski TA (2008). Chemokine receptor CXCR3 and its ligands CXCL9 and CXCL10 are required for the development of murine cerebral malaria.. Proc Natl Acad Sci U S A.

[13] Rudin W, Eugster HP, Bordmann G, Bonato J, Muller M (1997). Resistance to cerebral malaria in tumor necrosis factor-alpha/beta-deficient mice is associated with a reduction of intercellular adhesion molecule-1 up-regulation and T helper type 1 response.. Am J Pathol.

[14] Amante FH, Haque A, Stanley AC, Rivera Fde L, Randall LM (2010). Immune-mediated mechanisms of parasite tissue sequestration during experimental cerebral malaria.. J Immunol.

[15] McQuillan JA, Mitchell AJ, Ho YF, Combes V, Ball HJ (2011). Coincident parasite and CD8 T cell sequestration is required for development of experimental cerebral malaria.. Int J Parasitol.

[16] Senaldi G, Shaklee CL, Guo J, Martin L, Boone T (1999). Protection against the mortality associated with disease models mediated by TNF and IFN-gamma in mice lacking IFN regulatory factor-1.. J Immunol.

[17] Patel SN, Berghout J, Lovegrove FE, Ayi K, Conroy A (2008). C5 deficiency and C5a or C5aR blockade protects against cerebral malaria.. J Exp Med.

[18] de Kossodo S, Grau GE (1993). Profiles of cytokine production in relation with susceptibility to cerebral malaria.. J Immunol.

[19] Bagot S, Campino S, Penha-Goncalves C, Pied S, Cazenave PA (2002). Identification of two cerebral malaria resistance loci using an inbred wild-derived mouse strain.. Proc Natl Acad Sci U S A.

[20] Campino S, Bagot S, Bergman ML, Almeida P, Sepulveda N (2005). Genetic control of parasite clearance leads to resistance to Plasmodium berghei ANKA infection and confers immunity.. Genes Immun.

[21] Berghout J, Min-Oo G, Tam M, Gauthier S, Stevenson MM (2010). Identification of a novel cerebral malaria susceptibility locus (Berr5) on mouse chromosome 19.. Genes Immun.

[22] Ohno T, Nishimura M (2004). Detection of a new cerebral malaria susceptibility locus, using CBA mice.. Immunogenetics.

[23] Sauer JD, Sotelo-Troha K, von Moltke J, Monroe KM, Rae CS (2011). The N-ethyl-N-nitrosourea-induced Goldenticket mouse mutant reveals an essential function of Sting in the in vivo interferon response to Listeria monocytogenes and cyclic dinucleotides.. Infect Immun.

[24] Hong CJ, Tsai PJ, Cheng CY, Chou CK, Jheng HF (2010). ENU mutagenesis identifies mice with morbid obesity and severe hyperinsulinemia caused by a novel mutation in leptin.. PLoS One.

[25] Miller G, Neilan M, Chia R, Gheryani N, Holt N (2010). ENU mutagenesis reveals a novel phenotype of reduced limb strength in mice lacking fibrillin 2.. PLoS One.

[26] Fernandez L, Marchuk DA, Moran JL, Beier DR, Rockman HA (2009). An N-ethyl-N-nitrosourea mutagenesis recessive screen identifies two candidate regions for murine cardiomyopathy that map to chromosomes 1 and 15.. Mamm Genome.

[27] Chan ER, Lavender H, Li G, Haviernik P, Bunting KD (2009). An ENU-induced recessive mutation in Mpl leads to thrombocytopenia with overdominance.. Exp Hematol.

[28] Thomis DC, Gurniak CB, Tivol E, Sharpe AH, Berg LJ (1995). Defects in B lymphocyte maturation and T lymphocyte activation in mice lacking Jak3.. Science.

[29] Baptista FG, Pamplona A, Pena AC, Mota MM, Pied S (2010). Accumulation of Plasmodium berghei-infected red blood cells in the brain is crucial for the development of cerebral malaria in mice.. Infect Immun.

[30] North RJ, Jung YJ (2004). Immunity to tuberculosis.. Annu Rev Immunol.

[31] Simmons CP, Clare S, Ghaem-Maghami M, Uren TK, Rankin J (2003). Central role for B lymphocytes and CD4+ T cells in immunity to infection by the attaching and effacing pathogen Citrobacter rodentium.. Infect Immun.

[32] Higgins LM, Frankel G, Douce G, Dougan G, MacDonald TT (1999). Citrobacter rodentium infection in mice elicits a mucosal Th1 cytokine response and lesions similar to those in murine inflammatory bowel disease.. Infect Immun.

[33] Simmons CP, Goncalves NS, Ghaem-Maghami M, Bajaj-Elliott M, Clare S (2002). Impaired resistance and enhanced pathology during infection with a noninvasive, attaching-effacing enteric bacterial pathogen, Citrobacter rodentium, in mice lacking IL-12 or IFN-gamma.. J Immunol.

[34] WHO (2010).

[35] Lackner P, Part A, Burger C, Dietmann A, Broessner G (2009). Glatiramer acetate reduces the risk for experimental cerebral malaria: a pilot study.. Malar J.

[36] Morrell CN, Srivastava K, Swaim A, Lee MT, Chen J (2011). Beta interferon suppresses the development of experimental cerebral malaria.. Infect Immun.

[37] Franklin BS, Ishizaka ST, Lamphier M, Gusovsky F, Hansen H (2011). Therapeutical targeting of nucleic acid-sensing Toll-like receptors prevents experimental cerebral malaria.. Proc Natl Acad Sci U S A.

[38] Cabrales P, Zanini GM, Meays D, Frangos JA, Carvalho LJ (2011). Nitric Oxide Protection Against Murine Cerebral Malaria Is Associated With Improved Cerebral Microcirculatory Physiology.. J Infect Dis.

[39] Balachandar S, Katyal A (2011). Peroxisome proliferator activating receptor (PPAR) in cerebral malaria (CM): a novel target for an additional therapy.. Eur J Clin Microbiol Infect Dis.

[40] Cornejo MG, Boggon TJ, Mercher T (2009). JAK3: a two-faced player in hematological disorders.. Int J Biochem Cell Biol.

[41] Notarangelo LD, Mella P, Jones A, de Saint Basile G, Savoldi G (2001). Mutations in severe combined immune deficiency (SCID) due to JAK3 deficiency.. Hum Mutat.

[42] Rudin W, Favre N, Bordmann G, Ryffel B (1997). Interferon-gamma is essential for the development of cerebral malaria.. Eur J Immunol.

[43] Yanez DM, Manning DD, Cooley AJ, Weidanz WP, van der Heyde HC (1996). Participation of lymphocyte subpopulations in the pathogenesis of experimental murine cerebral malaria.. J Immunol.

[44] Hansen DS, Bernard NJ, Nie CQ, Schofield L (2007). NK cells stimulate recruitment of CXCR3+ T cells to the brain during Plasmodium berghei-mediated cerebral malaria.. J Immunol.

[45] Mitchell AJ, Hansen AM, Hee L, Ball HJ, Potter SM (2005). Early cytokine production is associated with protection from murine cerebral malaria.. Infect Immun.

[46] Hermsen C, van de Wiel T, Mommers E, Sauerwein R, Eling W (1997). Depletion of CD4+ or CD8+ T-cells prevents Plasmodium berghei induced cerebral malaria in end-stage disease.. Parasitology.

[47] Yanez DM, Batchelder J, van der Heyde HC, Manning DD, Weidanz WP (1999). Gamma delta T-cell function in pathogenesis of cerebral malaria in mice infected with Plasmodium berghei ANKA.. Infect Immun.

[48] Finley R, Weintraub J, Louis JA, Engers HD, Zubler R (1983). Prevention of cerebral malaria by adoptive transfer of malaria-specific cultured T cells into mice infected with Plasmodium berghei.. J Immunol.

[49] McCall MB, Roestenberg M, Ploemen I, Teirlinck A, Hopman J (2010). Memory-like IFN-gamma response by NK cells following malaria infection reveals the crucial role of T cells in NK cell activation by P. falciparum.. Eur J Immunol.

[50] Kudlacz E, Perry B, Sawyer P, Conklyn M, McCurdy S (2004). The novel JAK-3 inhibitor CP-690550 is a potent immunosuppressive agent in various murine models.. Am J Transplant.

[51] Meyer DM, Jesson MI, Li X, Elrick MM, Funckes-Shippy CL (2010). Anti-inflammatory activity and neutrophil reductions mediated by the JAK1/JAK3 inhibitor, CP-690,550, in rat adjuvant-induced arthritis.. J Inflamm (Lond).

[52] Frucht DM, Gadina M, Jagadeesh GJ, Aksentijevich I, Takada K (2001). Unexpected and variable phenotypes in a family with JAK3 deficiency.. Genes Immun.

[53] Roberts JL, Lengi A, Brown SM, Chen M, Zhou YJ (2004). Janus kinase 3 (JAK3) deficiency: clinical, immunologic, and molecular analyses of 10 patients and outcomes of stem cell transplantation.. Blood.

[54] Zhou YJ, Chen M, Cusack NA, Kimmel LH, Magnuson KS (2001). Unexpected effects of FERM domain mutations on catalytic activity of Jak3: structural implication for Janus kinases.. Mol Cell.

[55] Yamaoka K, Saharinen P, Pesu M, Holt VE, Silvennoinen O (2004). The Janus kinases (Jaks).. Genome Biol.

[56] Royer Y, Staerk J, Costuleanu M, Courtoy PJ, Constantinescu SN (2005). Janus kinases affect thrombopoietin receptor cell surface localization and stability.. J Biol Chem.

[57] Rane SG, Reddy EP (2000). Janus kinases: components of multiple signaling pathways.. Oncogene.

[58] O'Shea JJ, Gadina M, Schreiber RD (2002). Cytokine signaling in 2002: new surprises in the Jak/Stat pathway.. Cell.

[59] Balling R (2001). ENU mutagenesis: analyzing gene function in mice.. Annu Rev Genomics Hum Genet.

[60] Fortin A, Diez E, Rochefort D, Laroche L, Malo D (2001). Recombinant congenic strains derived from A/J and C57BL/6J: a tool for genetic dissection of complex traits.. Genomics.

[61] Gros P, Skamene E, Forget A (1981). Genetic control of natural resistance to Mycobacterium bovis (BCG) in mice.. J Immunol.

