# Supplementary figures and images for: An N-Ethyl-N-Nitrosourea (ENU)-Induced Dominant Negative Mutation in the JAK3 Kinase Protects against Cerebral Malaria
Source: PLoS One. 2012 Feb 21;7(2):e31012. doi: 10.1371/journal.pone.0031012 (PMC3283600; doi:10.1371/journal.pone.0031012)

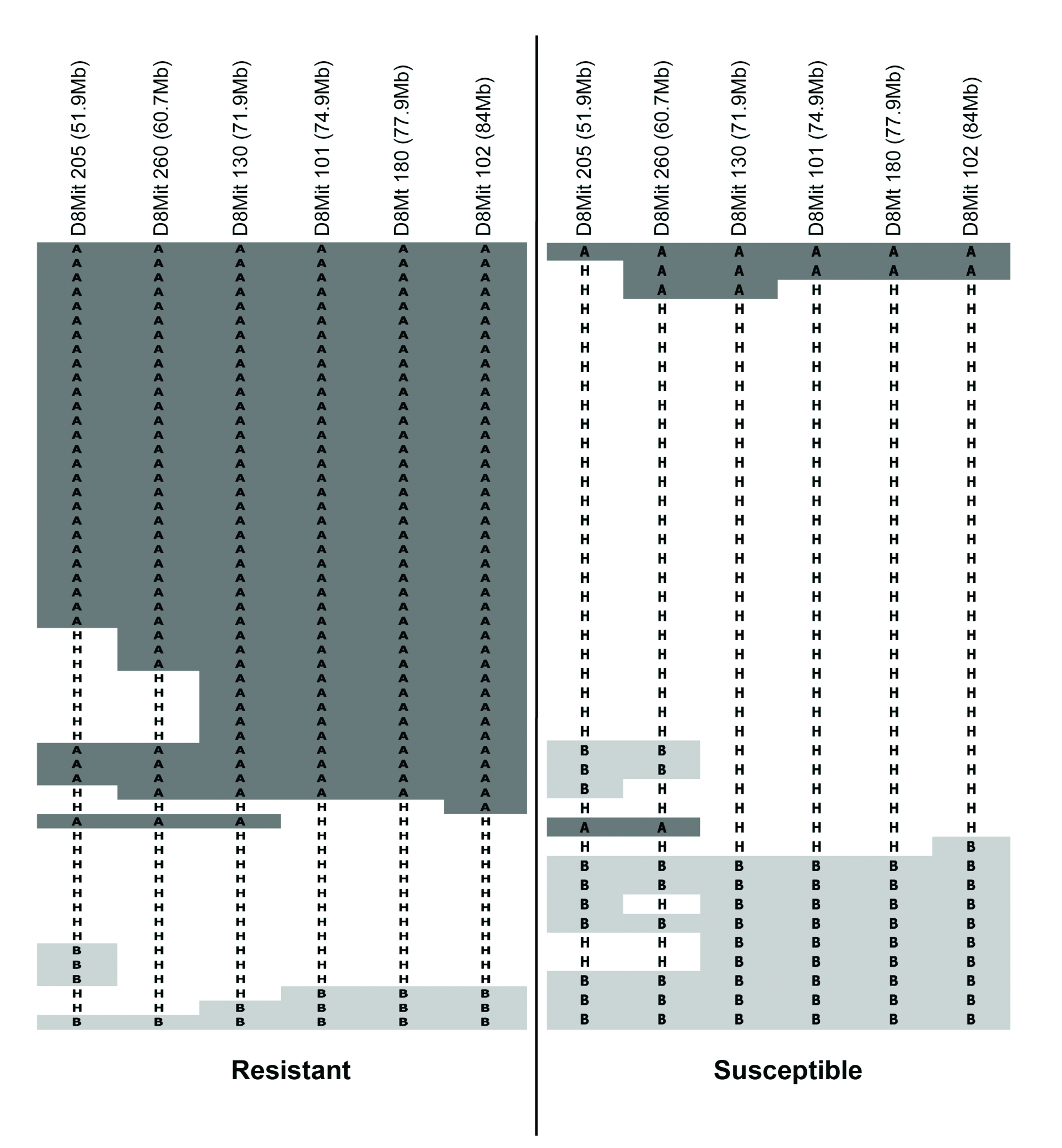

Supplement: Figure S1 — Haplotype map of F2 mice from pedigree 48 for the central portion of chromosome 8 (51.9–84 Mb). F2 mice generated by crossing the G1 male to 129S1 progenitors were genotyped for microsatellite markers (Mouse Genome Informatics Database; www.informatics.jax.org) in the 51.9–84 Mb interval, and were phenotyped for resistance and susceptibility to P. berghei induced CM. Each row represents the haplotype (A, homozygote B6; H, heterozygote; B, homozygote 129S1) of an individual mouse for the indicated polymorphic markers. (TIF) [file pone.0031012.s001.tif]
